# Participating in an International Stereotactic Radiotherapy Patient Registry: The Establishment of Data Collection Pathways

**DOI:** 10.7759/cureus.1413

**Published:** 2017-06-29

**Authors:** Aylin Yahya, Eva Arneric, Elizabeth Kernutt, Fiona Baldacchino, Claire Haworth, Mary-Anne Kedda, Colin Tang, Sean Bydder, Tammy Corica

**Affiliations:** 1 Radiation Oncology Clinical Trials and Research, Sir Charles Gairdner Hospital, Perth, Australia; 2 Department of Radiation Oncology, Sir Charles Gairdner Hospital, Perth, Australia

**Keywords:** data collection pathways, patient registry, cyberknife®

## Abstract

Aim

To describe data collection pathways and practical challenges experienced by an academic comprehensive cancer centre aiming to record clinical data for patients being treated with a novel radiotherapy treatment modality.

Methods

Various options to capture data from all patients treated with the CyberKnife Robotic Radiosurgery System at Sir Charles Gairdner Hospital (SCGH) in Western Australia were explored. An international multicenter web-based secure database established and maintained by the Radiosurgery Society the RSSearch® Patient Registry was selected. Data were collected and entered over four contiguous phases, with either opt-in or opt-out consent and the completion of Patient Reported Outcome questionnaires for specific sub-groups.

Results

Between April 2014 and June 2016, 461 patients at Sir Charles Gairdner Hospital were enrolled in the RSSearch® Patient Registry with the collection of over 17,500 data items. From 461 patients enrolled, 447 patients were treated with the CyberKnife Robotic Radiosurgery System. The majority of patients were treated for either a malignant primary (43.2%) or metastatic disease (39.4%). The establishment of matrix organisational processes for data collection led to the development of improved workflow patterns and data collection pathways.

Conclusions

This article describes the processes developed by a single centre to establish an efficient system for data collection and participation in an international registry. The opt-out approach was more efficient in terms of patient recruitment compared to the informed-consent method used in earlier phases. The experience of this single centre may help inform other institutions considering data collection options for assessments of new or novel treatments.

## Introduction

The CyberKnife Robotic Radiosurgery System is a state-of-the-art treatment modality with globally recognised benefits for the treatment of cancer and other diseases [1]. It is designed to deliver both cranial and extra-cranial stereotactic radiosurgery (SRS) and stereotactic body radiotherapy (SBRT). It has the ability to track skull, spine, suitable lung tumors and inserted marker seeds positions in real-time, to accurately deliver treatment [2]. CyberKnife is a unique device that integrates robotics with image-guidance technology and accurately targets to treat tumors and other lesions with high doses of radiation, either in skull or body while minimising exposure to nearby healthy tissues [3-4].

The Department of Radiation Oncology, Sir Charles Gairdner Hospital (SCGH) is the first site in Australia to implement the CyberKnife Radiosurgery System. The CyberKnife represents a substantial investment by the state, the hospital and the Radiation Oncology department, bringing with it the responsibility to ensure treatment is delivered as efficiently and as effectively as possible.

In order to achieve a high standard of quality healthcare and to inform local and international stakeholders of the utilisation of this device, it is essential to record treatment-related data [5-6]. Established databases such as registries were therefore explored as these were most likely to offer a more efficient and cost-effective data-capture process [7]. Registries provide real world data where effectiveness, safety and toxicity can be quickly measured and therefore lead to improved health care delivery [5, 8-9]. Although registries usually do not have the same limitations on inclusion criteria for data collection as clinical trials, they can play an important role in forming research questions [10-11]. Established patient databases can track clinical outcomes and also provide structure for future research studies [12]. In Australia, various national registries collecting outcome data on specific cancer streams already exist. The Prostate Cancer Registry is one example [13], however, the need for a comprehensive registry that covers all tumor streams treated with CyberKnife was required. It was also important to use an established registry developed with expert knowledge, to ensure capture of key clinical variables and components to reflect realistic and practical data collection, entry and reporting. Utilising an existing registry with well supported technical infrastructure was also a priority. The review process concluded that the international multi-institutional RSSearch® Patient Registry would best achieve the outcomes desired and participation commenced in April 2014.

The role of the RSSearch® Patient Registry

The RSSearch® Patient Registry (from now on will be referred to as the ‘Registry’) was established in 2006 by The Radiosurgery Society® (RSS), a multi-disciplinary non-profit organization focusing on advancing the science and clinical practice of radiosurgery. The Registry database meets all requirements to maintain system security, participant confidentiality and transmission of data. It is a secure, HIPAA (Health Insurance Portability and Accountability Act for privacy of electronic health care) compliant system that at the time of implementation by SCGH, was maintained by Advertek® Inc. (Louisville, KY), an incorporated, independent, third-party developer of web-based registries [14]. To date, the Registry contains more than 17,400 patient records worldwide. Local and international data in the Registry are available for local quality assurance (QA) and quality improvement (QI) reviews and health economics evaluations, research projects, international collaborations and publications. An annual subscription fee is required to participate in the Registry.

All potential CyberKnife patients are deemed eligible to be enrolled in the Registry. The Registry is designed to collect patient demographics, tumor/lesion characteristics, treatment locations, treatment plan and delivery information, toxicity and clinical outcomes including symptom control, lesion response, patient survival, and disease progression.

Data is entered as per individual institutional guidelines and data analyses can be conducted retrospectively. Each participating site has access to their own dataset and the de-identified aggregate data can be accessed by the International Registry administrator for QA and scientific publications [14]. Individual centres can export their own data from the Registry at any time into customised MS Excel® spreadsheets. Upon application to the Radiosurgery Society, individual sites may also access records from other facilities around the world. The Registry is listed on the clinicaltrials.gov website as “RSSearch patient™ Registry-Long Term Study of Use of SRS/SBRT” and identified as NCT01885299.

## Materials and methods

**Figure 1:**
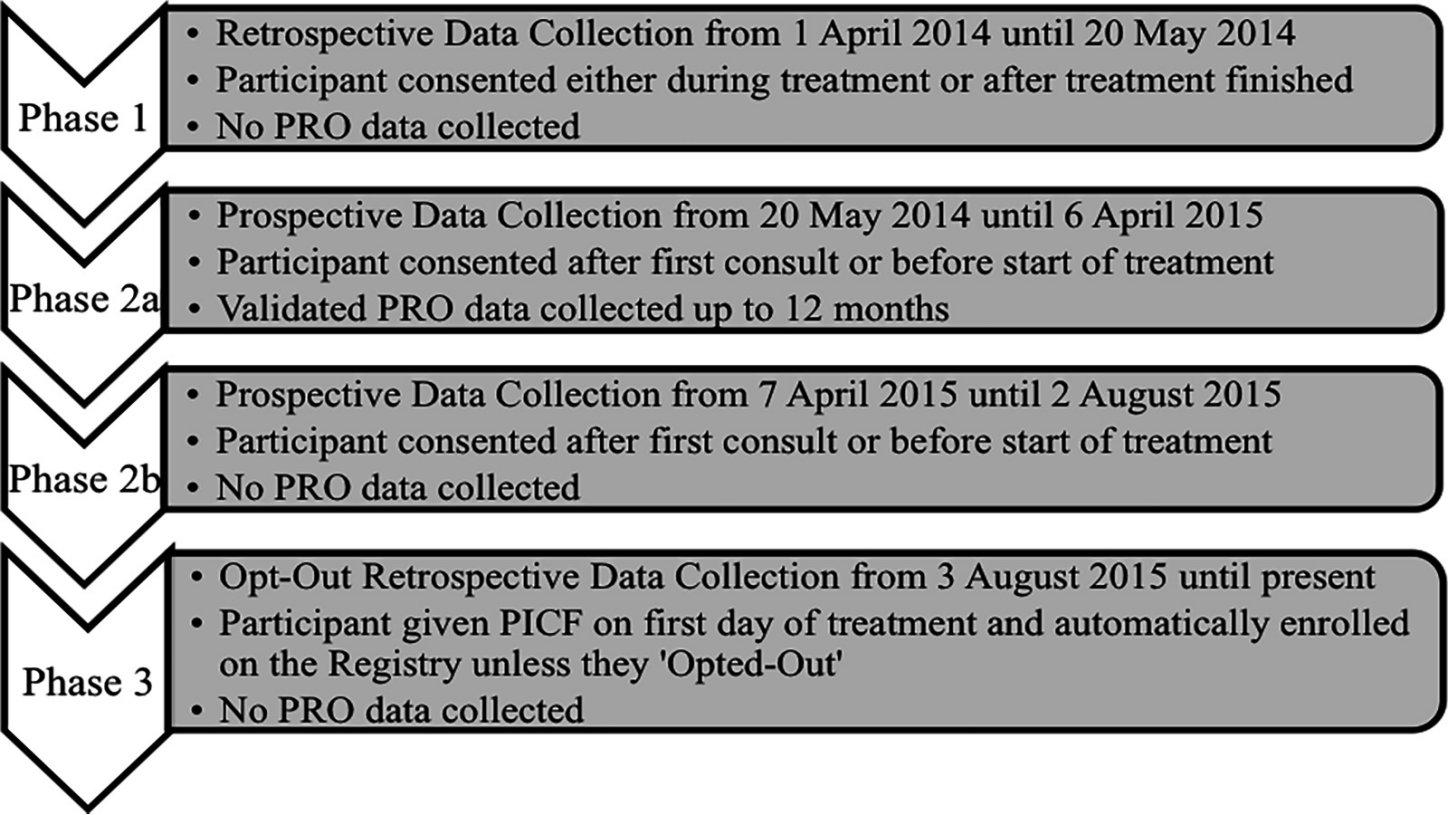
RSSearch® Patient Registry data collection phases at Sir Charles Gairdner Hospital (SCGH). PRO: Patient reported outcome.

SCGH commenced treating patients with CyberKnife in April 2014. Institutional Ethics approval allowed CyberKnife patients over the age of 18 to be approached by research staff to request permission for their data to be included in the Registry. Implementation of the Registry followed the model used for most other research studies: A screening log was designed to record all potential CyberKnife patients who could be considered for the registry; Data collection tools and schedules were developed to facilitate data collection at the specified time points; Paper-based Case Report Forms (CRFs) were designed to capture required data fields. Individual patient folders were created to house relevant source data. Since joining the RSSearch® Patient Registry, data collection has evolved through four phases (Figure 1).

Phase 1

The first phase of data collection was retrospective, for patients who had already commenced or had recently completed CyberKnife treatment. It was important to include these patients in order to capture all patients from the introduction of CyberKnife treatment. Patients were given a patient information sheet to read and provide written informed consent, followed by relevant data being abstracted from medical records onto custom-made CRFs and then entered into the Registry. A sticker-system was put in place to enable collection of future follow-up data. Patient reported outcomes (PROs) were not collected during this phase because patients had already started or finished their treatment and it was not feasible to collect such data retrospectively.

Phase 2a

In Phase 2a, patient data was collected prospectively, enabling the collection of PROs. Written informed consent was obtained from all eligible patients prior to the start of treatment (usually at their CT planning appointment). Data collection included completion of clinician and/or trial coordinator-completed CRFs. Patients were asked to complete PROs prior to treatment (baseline) and at three time-points post-treatment (6-12 weeks, 6 months and 1 year). PROs were collected to assess functional health at baseline and post CyberKnife treatment. Validated, internationally recognised and free of charge tools were chosen, The European Organisation for Research and Treatment of Cancer (EORTC) QLQ-C30 and the Sickness Impact Profile (SIP). The EORTC has been used extensively in cancer research and has also been used elsewhere in CyberKnife studies for lung and pancreatic cancers [15-16]. The QLQ-C30 was supplemented by disease-specific modules to provide further insight into specific toxicities associated with treatment to that anatomical region. Modules utilised were breast, lung, brain, head and neck, oesophageal, gastric, hepatocellular, prostate, colorectal, liver metastases and bone metastases. The SIP was administered to patients who were being treated for non-malignant diseases, as it measures the change in behaviour as a consequence of illness [17]. The entry of PRO data was not easily supported by the Registry, therefore, an in-house stand-alone MS Access® Database was developed. The collection of PRO data ceased after one year due to resourcing issues and uncertainty regarding standardisation of time-points and instruments being used across all tumor sites.

Phase 2b

SCGH screening records showed that 10% of potential CyberKnife participants declined to participate in the Registry. Reasons for non-participation included concerns over the additional burden of the PRO questionnaires. In the best interests of resourcing and data quality at the time, it was decided that PROs would no longer be collected, giving rise to Phase 2b.

Phase 3

Recruitment of new patients, together with the follow-up requirements for existing patients, continued to put pressure on the existing resources of the research unit. Further measures to ensure all new patients were invited to participate and that their follow-up data could be captured were investigated. As per the National Statement, section 2.3.6, “research activity is likely to be compromised if the participation rate is not near complete, and the requirement for explicit consent would compromise the necessary level of participation”[18]. There was also evidence that good quality registries require near-complete data collection, which can usually be achieved with an opt-out approach [5]. This approach was implemented in Phase 3, the current data collection phase, whereby patients are given an Opt-Out Participant Information Sheet on their first day of treatment. For patients not opting out, data are collected from the patient’s medical records retrospectively at non-specified time points and usually when patients return for a routine visit. No on-going research coordinator–patient contact is required after the initial registry discussion and no PROs are collected. Patients who decide against participation are required to complete the opt-out form so that no retrospective data collection is performed. To enable sustainable data collection, a ‘minimum dataset registry sticker’ has been created and is included with the medical record, to capture follow-up treatment outcome data at each patient’s follow-up appointment, which are then entered into the Registry.

## Results

**Figure 2:**
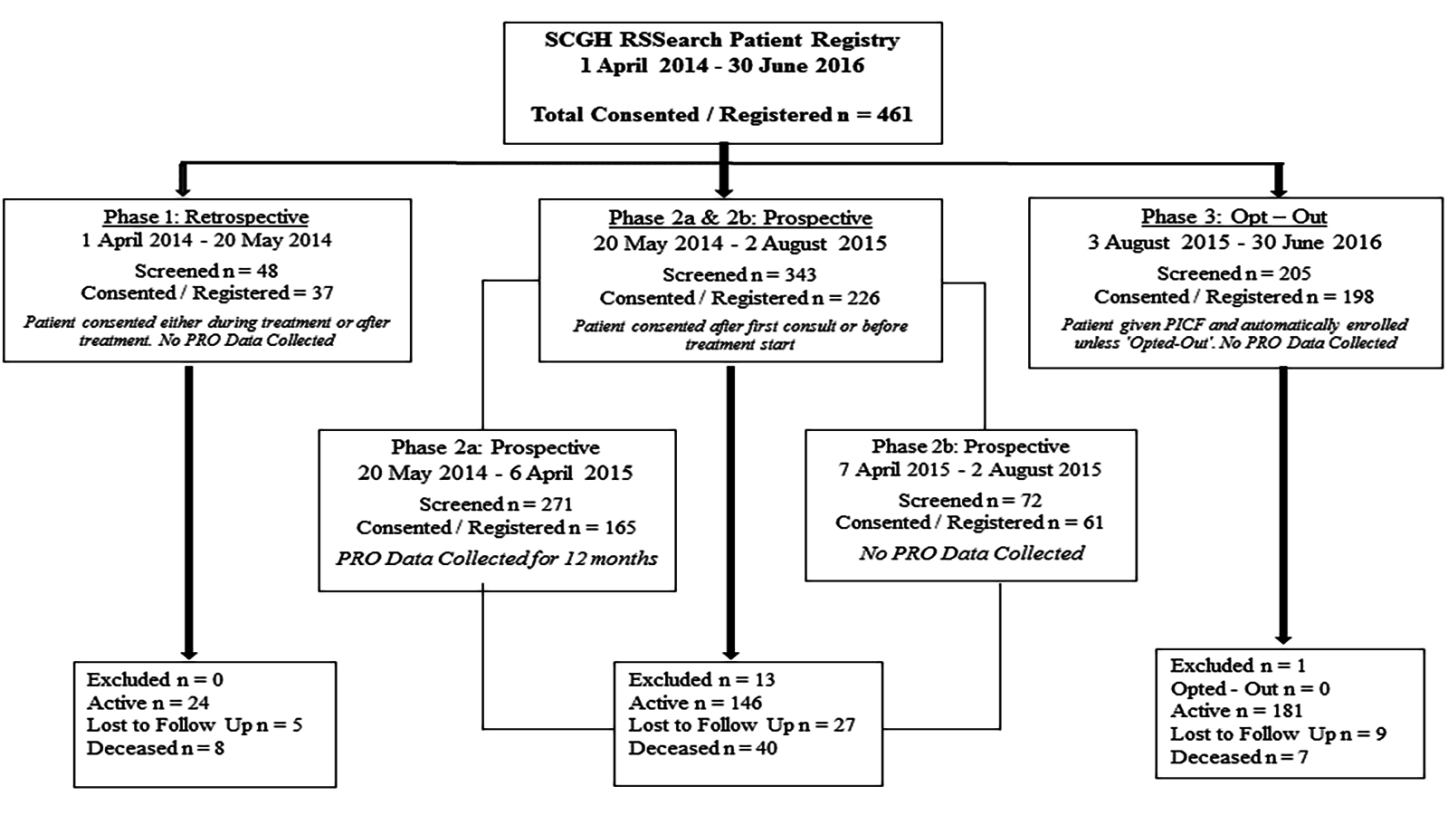
Number of participants enrolled in the RSSearch® Patient Registry. PRO: Patient reported outcome; SCGH: Sir Charles Gairdner Hospital.

Overall, 461 patients were registered in the RSSearch® Patient Registry at SCGH between April 2014 and June 2016. At the end of 2015, SCGH was ranked third in recruitment out of 42 international centres participating in the Registry [19]. Currently, there are 351 patients on active follow-up post CyberKnife treatment, 41 are considered lost to follow-up and 55 deceased. Fourteen were excluded from the Registry for various reasons, e.g., CyberKnife treatment could not be given as technically impossible or not clinically appropriate, or the patient declined treatment. Of 198 patients approached during the Phase 3 opt-out phase, no one has opted-out (Figure 2).

Of the 461 patients enrolled in the Registry, 447 patients were treated with the CyberKnife Robotic Radiosurgery System. The majority were male, Caucasian, aged between 50 and 70 years and resided in a metropolitan area, with two patients of Australian Aboriginal heritage (Table 1).

**Table 1:** Patient characteristics and demographics.

Total number of patients (n = 447)	(n)	(%)
Gender		
Male	288	64.4%
Female	159	35.6%
Age groups		
<50	51	11.4%
50-70	243	54.4%
>70	153	34.2%
Area of residence		
Metropolitan	322	72.0%
Rural	125	28.0%
Ethnicity		
Caucasian	414	92.6%
Asian	14	3.1%
Other (including two Australian Aboriginal patients)	11	2.5%
Multiracial	3	0.6%
African-American	2	0.5%
Pacific-Asian Islander	2	0.5%
Hispanic	1	0.2%

Eight percent of patients were treated in Phase 1, 48% in Phase 2a and 2b and 44% in the opt-out Phase 3 (Table 2).

**Table 2:** Patients treated per phase.

Total number of patients (n = 447)	(n)	(%)
Phase 1	37	8.3%
Phase 2a and 2b	213	47.7%
Phase 3	197	44.1%

The main treatment sites were brain, cranial nerve and spinal cord (28.9%), lung and bronchus (25.3%), prostate (21.5%) and bones and joints (5.1%). The remaining 19% had treatment in other locations, mainly including liver and lymph nodes (Table 3).

**Table 3:** Clinical characteristics.

Patients treated per site (n = 447)	(n)	(%)
Brain, cranial nerve and spinal cord	129	28.9%
Lung and bronchus	114	25.5%
Prostate	96	21.5%
Bones and joints	23	5.1%
Other	85	19.0%

The majority of patients were treated for malignant primary tumors (43.2%) or metastatic tumors (39.4%) (Table 4).

**Table 4:** Lesion characteristics and most common lesion locations.

All lesions (n = 447)		(n)	(%)
Benign tumor		32	7.2%
	Brain, cranial nerve and spinal cord	22	68.8%
	Accessory, sinuses, middle and inner ear	7	21.9%
	Pituitary	1	3.1%
	Pharynx	1	3.1%
	Other nervous system	1	3.1%
Malignant primary tumor		193	43.2%
	Bones and joints	1	0.5%
	Brain, cranial nerve and spinal cord	10	5.2%
	Kidney	5	2.6%
	Liver	13	6.7%
	Lung and bronchus	59	30.6%
	Pancreas	5	2.6%
	Prostate gland	96	49.7%
	Renal pelvis, ureter	3	1.6%
	Trachea	1	0.5%
Metastatic tumor		177	39.6%
	Adrenal gland	3	1.7%
	Bones and joints	21	11.9%
	Brain, cranial nerve and spinal cord	74	41.8%
	Cerebellum	1	1.4%
	Oesophagus	1	0.6%
	Gallbladder and extrahepatic bile ducts	1	0.6%
	Kidney	2	1.1%
	Liver	12	6.8%
	Lung and bronchus	49	27.7%
	Lymph nodes	11	6.2%
	Mediastinum	1	0.6%
	Orbit and lacrimal gland (Excl. retina, eye, NOS)	1	0.6%
Recurrent primary tumor		37	8.3%
	Accessory, sinuses, middle and inner ear	2	5.4%
	Bones and joints	2	5.4%
	Brain, cranial nerve and spinal cord	15	40.5%
	Eye, NOS	1	2.7%
	Kidney	1	2.7%
	Liver	2	5.4%
	Lung and bronchus	6	16.2%
	Lymph nodes	1	2.7%
	Mediastinum	1	2.7%
	Nasopharynx	1	2.7%
	Pituitary	1	2.7%
	Prostate gland	1	2.7%
	Retroperitoneum and peritoneum	1	2.7%
	Tongue	1	2.7%
	Urinary bladder	1	2.7%
Functional disease		3	0.7%
	Brain, cranial nerve and spinal cord	3	100.0%
Arterio-venous malformation		5	1%
	Brain, cranial nerve and spinal cord	5	100%

Outcomes

The Registry provides an invaluable data source for clinicians to review treatment outcomes for various tumor locations. For example, an internal audit was conducted for all patients with primary hepatocellular carcinoma (HCC) disease treated with CyberKnife between April 2014 and June 2015. A similar audit has been conducted for prostate cancer and has been published [20]. Internal audits are also conducted every six months for in-house QA and QI activities, investigating data completeness and consistency in data entry techniques across local users. Findings are discussed with all users and improvements are made where necessary. The Radiosurgery Society® requested the inclusion of SCGH prostate data in a multi-centre publication [21].

## Discussion

Challenges

Being the world’s third highest recruiting centre for the RSSearch® Patient Registry after a short period of operation is a great achievement [16], however, a number of hurdles had been overcome to achieve this, with each data collection phase bringing its own unique challenges.

Phases 1 and 2 were resource-intensive and very time consuming for data collection and patient follow-up: each patient had to be monitored as to when they were coming in for clinic appointments, so that CRFs and PROs could be completed at the specified time-points. Another challenge was having standardised data collection time-points for follow-up visits and collection of PROs across all disease sites, since follow-up visits are usually at the clinician’s discretion. These challenges resulted in the opt-out phase being introduced. Phase 3 ensured data from all CyberKnife treated patients could continue to be collected and entered into the Registry, but with a saving of up to two-thirds of the time invested by the research unit staff.

There were also some challenges with the Registry itself. Inconsistencies between Australian and American terminology made it difficult to enter some data accurately into the US-based Registry, including date formats, anatomical terminology, baseline measures, histopathology and units of measure. There are also a few ways data can be entered when an existing Registry patient undergoes treatment on subsequent locations. This requires discussion at the site level to standardise the method that is to be used across all such patients. A further challenge is despite the availability of a useful data dictionary, the Registry does not offer paper-based CRFs. This is in part because the Registry allows individual sites to choose what extent of data they wish to collect, hence sites wishing to use paper-based data collection instruments need to develop their own. This exercise proved time-consuming, but worthwhile given that paper-based CRFs work well locally.

A limiting factor of the Registry was the inability to directly enter PRO data. As such, a purpose-built local PRO database was commissioned, however future versions of the RSS Patient Registry may address this. The Registry also lacks the ability to map directly with other databases, making the analysis between registry data and PRO data onerous. Internal audits flagged the complexities of data linkage if specified time points are not clearly made in the Registry, which was another resource-intensive process to rectify.

Discussion

This paper reports the experiences of the first Australian single-centre collecting long-term clinical registry data for patients receiving CyberKnife treatment. Participation in the Registry was possible due to a well-resourced Research Unit. However, despite a well-developed system for sharing the work-load between staff, and having one designated local Registry administrator, the Registry work-load competed with other clinical trial and research responsibilities of the research unit. The establishment of matrix organisational processes for a co-ordinated approach, with different team members taking responsibility for different steps at different time points, was therefore undertaken.

Participation in the RSSearch® Patient Registry has enabled SCGH to collect clinical data for all patients treated at the first CyberKnife facility in Australia. It has allowed clinicians and research staff to access patient data from the Registry in customised MS Excel® spreadsheets and to link this data with patient-reported outcomes. As a data custodian of the RSSearch® Patient Registry, opportunities exist to access more than 17,400 patient records from other facilities around the world as well as being involved in health economics evaluations and other research projects, international collaborations and publications. Registry participation also provides the opportunity to evaluate the effectiveness of local management practices and assist with the development of treatment guidelines with the potential to guide the development of future CyberKnife clinical trial protocols.

The Radiosurgery Society has recently announced the launch of an updated RSSearch Patient Registry using the cloud-based VisionTree Optimal Care 360 platform [22] and our centre will continue the collection of screening, treatment and follow-up data utilizing the new platform. The new platform will facilitate the collection of more comprehensive data and participation in future research collaborations.

## Conclusions

The successful implementation of the RSSearch® Patient Registry, coupled with an active and dedicated approach to implementing and maintaining a sustainable system for data collection for patients treated with CyberKnife® at SCGH, has led to a robust dataset of over 400 patients treated between April 2014 and June 2016. This data can now be used for local quality improvement activities as well as international collaborative research. The current opt-out system in place has highlighted the benefits of this NHMRC-approved approach for clinical registries, in terms of both data completeness and resource optimisation. With the data collection tools and systems already in place, future CyberKnife research will be much easier to implement at SCGH. The experience of this single centre may also help to inform other institutions considering data collection options for new or novel treatments.
